# Amyloid-β_42_ stimulated hippocampal lactate release is coupled to glutamate uptake

**DOI:** 10.1038/s41598-022-06637-2

**Published:** 2022-02-17

**Authors:** Erin R. Hascup, Lindsey N. Sime, Mackenzie R. Peck, Kevin N. Hascup

**Affiliations:** 1 Department of Neurology, Dale and Deborah Smith Center for Alzheimer’s Research and Treatment, Neurosciences Institute, Southern Illinois University School of Medicine, P.O. Box 19628, Springfield, IL 62794-9628 USA; 2 Department of Pharmacology, Southern Illinois University School of Medicine, Springfield, IL USA; 3 Department of Medical Microbiology, Immunology and Cell Biology, Southern Illinois University School of Medicine, Springfield, IL USA

**Keywords:** Diseases of the nervous system, Synaptic transmission, Dementia

## Abstract

Since brain glucose hypometabolism is a feature of Alzheimer’s disease (AD) progression, lactate utilization as an energy source may become critical to maintaining central bioenergetics. We have previously shown that soluble amyloid-β (Aβ)_42_ stimulates glutamate release through the α7 nicotinic acetylcholine receptor (α7nAChR) and hippocampal glutamate levels are elevated in the APP/PS1 mouse model of AD. Accordingly, we hypothesized that increased glutamate clearance contributes to elevated extracellular lactate levels through activation of the astrocyte neuron lactate shuttle (ANLS). We utilized an enzyme-based microelectrode array (MEA) selective for measuring basal and phasic extracellular hippocampal lactate in male and female C57BL/6J mice. Although basal lactate was similar, transient lactate release varied across hippocampal subregions with the CA1 > CA3 > dentate for both sexes. Local application of Aβ_42_ stimulated lactate release throughout the hippocampus of male mice, but was localized to the CA1 of female mice. Coapplication with a nonselective glutamate or lactate transport inhibitor blocked these responses. Expression levels of SLC16A1, lactate dehydrogenase (LDH) A, and B were elevated in female mice which may indicate compensatory mechanisms to upregulate lactate production, transport, and utilization. Enhancement of the ANLS by Aβ_42_-stimulated glutamate release during AD progression may contribute to bioenergetic dysfunction in AD.

## Introduction

Alzheimer’s disease (AD) is the most prevalent form of dementia and is characterized by progressive anterograde amnesia, hippocampal atrophy, and eventual death. The majority of approved medications treat symptomatic progression, while emerging options are purported to modify disease outcome. Currently, biomarkers indicate advanced AD progression which limits clinical diagnosis to after memory deficits and neuronal loss. A better understanding of the neurochemical changes during the initial stages of AD development are needed to improve therapeutic outcome.

The hallmark pathognomonic signs of AD include senile plaques composed of aggregated amyloid-β (Aβ)_42_ and neurofibrillary tangles consisting of hyperphosphorylated tau. Although plaque accumulation is believed to occur earlier in disease progression^1,2^, soluble isoforms of Aβ_42_ are neurotoxic and may precipitate AD pathology^3–6^. Our laboratory has previously demonstrated that soluble Aβ_42_ stimulates presynaptic glutamate release through activation of the alpha 7 nicotinic acetylcholine receptor (α7nAChR)^7^. As AD progresses soluble Aβ_42_ concentrations increase leading to persistent activation of these receptors that chronically elevate extracellular glutamate levels^8^. This increased glutamate continuously  activates postsynaptic receptors thereby dampening responses from physiological stimuli, which is hypothesized to cause the cognitive and functional decline observed in AD^9^. Accordingly, symptomatic effects are observed with memantine, a partial NMDA receptor antagonist, through modulation of the glutamatergic tone.

Glutamatergic signaling is terminated by uptake into Na^+^-dependent, high-affinity excitatory amino acid transporters (EAAT) located on glia^10^. The co-transport of glutamate and Na^+^ by EAAT into astrocytes activates the Na^+^/K^+^ ATPase to reestablish the resting membrane potential^11^. The ATP consumed by this pump is replenished by astrocytic glycogenolysis^12^ and glycolysis^13^ leading to lactate generation from glial glutamate uptake^14^. Monocarboxylate transporters (MCT) shuttle lactate out of astrocytes (MCT1 and 4) and into neurons (MCT2) where it is converted to pyruvate for use in mitochondrial oxidative phosphorylation^15^. This process, referred to as the astrocyte-neuron lactate shuttle (ANLS), couples neurotransmission and neuroenergetics^11,14,16^. The ANLS provides lactate to neurons for an additional oxidative substrate^15^ during increased energy demands, which is also essential for long-term memory consolidation^12,17^. Conversely, others have proposed the glucose sparing concept that asserts astrocytic utilization of glycogen spares their need for blood derived glucose^18^. This would provide neurons with enough glucose to meet energy demands during neurotransmission and memory consolidation^19^.

Although both neurons and astrocytes increase glycolysis after stimulation^20,21^, lactate is provided to neurons from astrocytes as a means to support increased energy demands^22^. However, brain glucose hypometabolism and vascular dysfunction are hallmark features of AD progression and suggests an inadequate supply of blood derived glucose to meet energy requirements. Lactate utilization as an energy source may become critical to maintaining the electrochemical gradient during AD progression. For example, lactate levels are elevated in the brains of amnestic mild cognitive impairment patients^23^, and in the cerebrospinal fluid of AD patients^24–26^. Cortical and hippocampal lactate levels increase with disease progression in the APP/PS1 transgenic mouse model of AD^27^. Based upon our previous research^7,8^, we hypothesized that the elevated extracellular lactate may result from increased Aβ_42_ stimulated glutamate release and subsequent activation of the ANLS. To test this hypothesis, we utilized an enzyme-based microelectrode array (MEA) selective for measuring tonic and phasic extracellular lactate^28,29^. Changes in hippocampal extracellular lactate levels were measured in male and female C57BL/6J mice upon local application of Aβ_42_ with and without EAAT and MCT inhibitors to examine the glutamate-lactate interplay as it relates to AD.

## Results

### Hippocampal basal and transient lactate analysis

**Figure 1:**
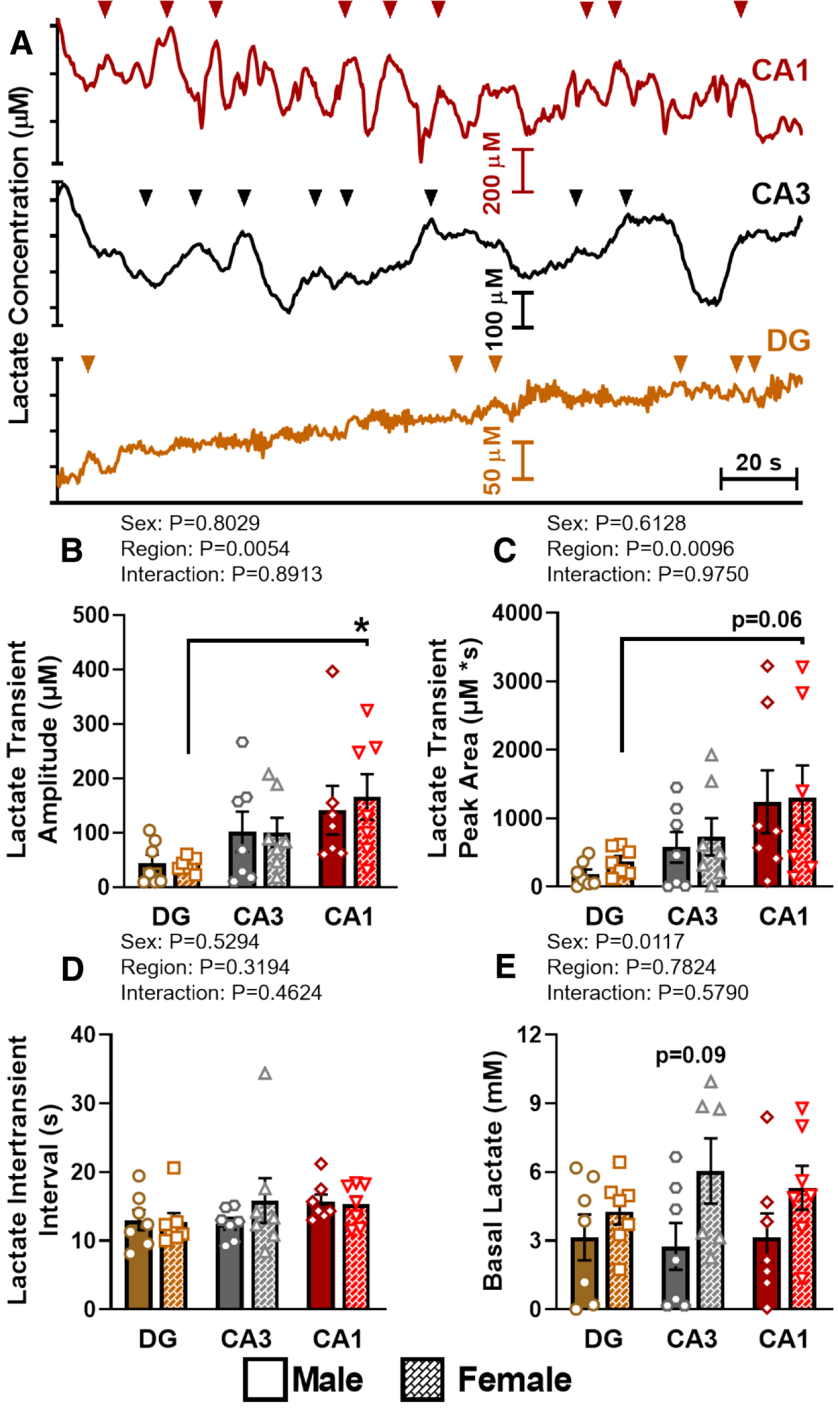
Transient and basal hippocampal lactate. Representative transient lactate release (**A**) is shown in the DG (brown), CA3 (black), and CA1 (red) of female C57BL/6 mice. Note the different scales of the ordinate y-axis in each subregion and color-coordinated triangles identify transient release events. The average lactate transient amplitude (**B**), peak area (**C**) and intertransient interval (**D**) were averaged for each hippocampal region and sex. *p < 0.05; n = 6–7. A measurement without detectable lactate transients was used to calculate basal lactate levels (**E**).

We have previously reported spontaneous or transient release of tonic glutamate in the prefrontal cortex and hippocampus^30,31^. These signals were tetrodotoxin dependent, a sodium channel blocker, supporting release from synaptic signaling^31^. Based on these previous studies and the ANLS hypothesis, we expected to observe spontaneous tonic fluctuations in lactate signaling. Once a stable baseline was reached, MEAs were used to measure extracellular lactate levels for 10 min in each hippocampal subregion prior to pressure ejection recordings. Transient lactate release was observed in all hippocampal subregions and both sexes of C57BL/6J mice. Figure 1A shows representative DG, CA3, and CA1 traces from female mice. The average transient amplitude (Fig. 1B) was similar within a subregion between sexes, but was significantly different across hippocampal subregions. The largest amplitudes occurred within the CA1, followed by the CA3, and then DG of both male and female mice. Likewise, lactate transient peak area (Fig. 1C) varied across subregions with the largest in the CA1 that declined across the CA3 and DG. No differences between sexes were observed for any hippocampal subregion. The elevated transient release of lactate in the CA1 region may be in response to increased energy demands required by glutamatergic pyramidal neurons compared to granule cells types located in the DG. Despite differences in amplitude, the intertransient interval (Fig. 1D) was similar across all three subregions and sex differences were also not observed. After transient lactate analysis, basal levels were calculated without the detection of transient lactate release. Basal lactate (Fig. 1E) was increased in female C57BL/6 mice particularly within the CA3 hippocampal subregion.

### Soluble Aβ_42_ stimulates lactate release that is attenuated by blocking glutamate clearance

**Figure 2:**
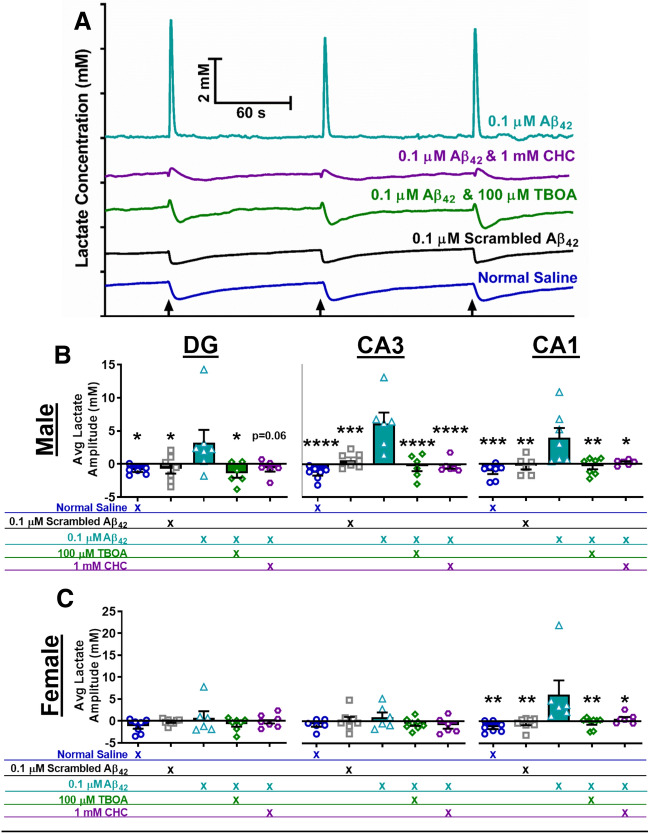
Efflux and inhibition of Aβ_42_ stimulated lactate release. Representative traces from application of normal saline (blue), 0.1 µM Scrambled Aβ_42_ (black), 0.1 µM Aβ_42_ (teal), 0.1 µM Aβ_42_ and 100 µM TBOA (green), and 0.1 µM Aβ_42_ and 1 mM CHC (purple) are shown in (**A**). Traces are offset on the ordinate to clearly show lactate dynamics and black arrows indicate time point of pressure ejection. Average maximal change in lactate levels from baseline after application of each compound for each hippocampal subregion of male (**B**) and female (**C**) C57BL/6 J mice. The legend under the graph indicates the compounds that were locally (co)applied. *p < 0.05, **p < 0.01, ***p < 0.001, ****p < 0.0001; n = 6–8.

After transient and basal lactate measurements, pressure ejections studies were performed. All compounds were tested in vitro to confirm they were not electrochemically active at our recording potential prior to local application in vivo. For each compound, consistent volumes between 25 and 75 nl were locally applied into the DG, CA3, and CA1 and no differences were observed between compounds within a brain region (Table 1). The 0.1 µM Aβ_42_ concentration was chosen because this elicited the maximal change in hippocampal glutamate release as shown in our previous research^7^. Representative lactate traces from local application in the CA1 of male C57BL/6J mice for all compounds are shown in Fig. 2A. Application of Aβ_42_ elicited robust and reproducible lactate signals. The average change in lactate amplitude from baseline for each hippocampal region is shown for male (2B) and female (2C) mice. In male mice, local application of 0.1 µM Aβ_42_ elicited lactate release in all hippocampal regions that was significantly higher compared to saline (vehicle control) and 0.1 µM scrambled Aβ_42_ (negative peptide control). In female mice, lactate release from local application 0.1 µM Aβ_42_ was significantly higher in the CA1 compared to saline and 0.1 µM scrambled Aβ_42_, but these effects were not observed in either the DG or CA3.

**Table 1:** Average pressure ejected volumes.

	Hippocampal region	Compound volume ejected (nl)					P value
		Saline	Scrambled Aβ_42_	Aβ_42_	TBOA	CHC	
Male C57BL/6J	DG	38 ± 3	39 ± 3	43 ± 5	36 ± 2	38 ± 4	0.6218
	CA3	37 ± 2	36 ± 3	42 ± 3	33 ± 2	35 ± 3	0.1270
	CA1	37 ± 1	34 ± 3	40 ± 3	37 ± 2	39 ± 3	0.5401
Female C57BL/6J	DG	38 ± 1	38 ± 3	32 ± 2	38 ± 2	40 ± 1	0.1671
	CA3	38 ± 3	38 ± 4	46 ± 11	37 ± 3	39 ± 2	0.7447
	CA1	38 ± 2	37 ± 3	43 ± 5	36 ± 3	39 ± 1	0.6270

Volumes are shown in mean ± SEM for application of each compound for all brain regions and sexes along with the corresponding P value from a one-way ANOVA.

Abbreviations -* Aβ* amyloid-β, *TBOA* dl-threo-β-Benzyloxyaspartic acid, *CHC* 2-cyano-3-(4-hydroxyphenyl)-2-propenoic acid.

Our previous research showed soluble Aβ_42_ elicited glutamate release through sodium-dependent α7nAChR mechanisms supporting a role for presynaptic release. To determine if Aβ_42_ mediated lactate release was coupled to glutamate uptake, 0.1 µM Aβ_42_ was coapplied with 100 µM dl-threo-β-Benzyloxyaspartic acid (TBOA). TBOA is a competitive, non-transportable blocker of EAAT what we have previously shown to inhibit glutamate clearance from the extracellular space at these concentrations^32^. Coapplication of Aβ_42_ with TBOA significantly attenuated the lactate release in all hippocampal subregions of male C57BL/6 J mice (Fig. 2B) and in the CA1 of female mice (Fig. 2C). Next, we coapplied 0.1 µM Aβ_42_ with 1 mM 2-cyano-3-(4-hydroxyphenyl)-2-propenoic acid (CHC), a MCT inhibitor that has been shown to block glial lactate efflux at this concentration^33^. Coapplication of Aβ_42_ with CHC also blunted extracellular lactate levels in the hippocampus of C57BL/6J male and the CA1 region of female mice (Fig. 2B,C). These data support the increased extracellular lactate levels from local application of Aβ_42_ are coupled to clearance of glutamate from the extracellular space through EAATs and efflux from MCT.

### mRNA expression of glutamate and lactate transporters

**Figure 3:**
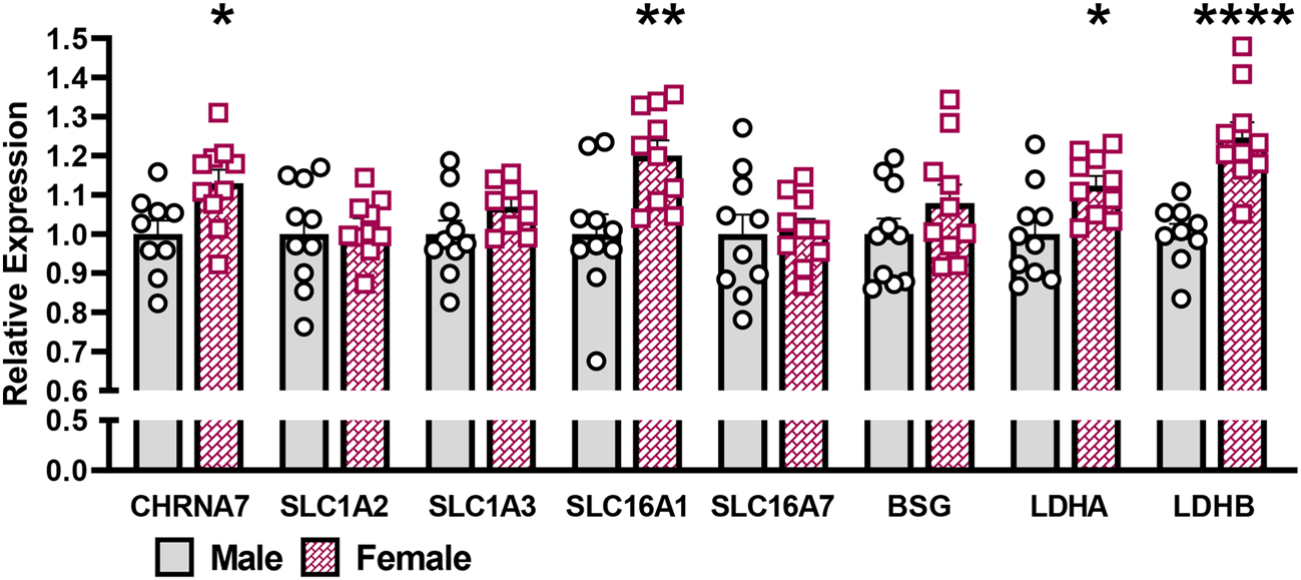
Hippocampal gene expression. Relative hippocampal mRNA expression levels of genes involved in the ANLS from male (gray) and female (purple) C57BL/6J mice. *p < 0.05, **p < 0.01, ****p < 0.0001; n = 9–10.

To determine if sex differences in lactate release were due to varying expression levels of genes involved with the ANLS, RT-PCR was conducted on tissue from a separate, naïve, cohort of male and female mice (Fig. 3). CHRNA7 expression levels were increased in female C57BL/6J mice while glial EAATs (SLC1A2 and SLC1A3) were similar between sexes. This suggests enhanced Aβ_42_-stimulated glutamate release without a change in the glutamate clearance in female mice. Next we examined expression levels involved with lactate production or utilization. Lactate dehydrogenase (LDH) is a tetramer consisting of either LDHA or LDHB subunits to form LDH-5 and LDH-1, respectively. In glial cells, homomeric LDHA catalyzes the reduction of pyruvate to lactate, while in neurons the homomeric LDHB isoform drives oxidation of lactate to pyruvate for oxidative phosphorylation^34^. Expression of both isoforms was increased in female mice indicating enhanced glial lactate production and neuronal utilization, respectively. While no sex differences were observed in neuronal SLC16A7 expression, we observed glial SLC16A1 expression was increased in female C57BL/6J mice. BSG is known to chaperone glial MCT1 and regulate basal and glutamate evoked lactate release through this transporter^35^. No BSG expression differences were observed between sexes.

## Discussion

Glucose enters the brain through transporters located on astrocytic endfeet that cover the surface of blood vessels. Glucose metabolism is the predominant fuel source for the brain serving as a substrate not only for ATP production in aerobic glycolysis but also providing the carbon backbone for maintaining the neurotransmitter pools of glutamate and γ-aminobutyric acid. This ATP production is necessary to reestablish the resting membrane potential for neuronal signaling which accounts for ~ 70% of the brain’s energy expenditure with excitatory neurons accounting for 80–85% of this usage^36^. Lactate is a byproduct of aerobic glycolysis and during periods of increased neuronal activity, can serve as an additional energy substrate through mitochondrial oxidative phosphorylation^11^. In this process, neuronally released glutamate is cleared from the extracellular space through EAAT located on glia. Uptake of glutamate is coupled to glucose transport into astroglia and a subsequent increase in glycolysis. The lactate formed is shuttled from astrocytes to neurons through MCT thereby coupling glutamatergic neurotransmission with bioenergetics to support synaptic signaling.

Once thought of as a metabolic waste product, the role of lactate in brain health and disease has garnered much attention over the last two decades. During acute neurotrauma, such as with traumatic brain injury or cerebral ischemia, astrocytic release of lactate provides neuroprotection^37^. Lactate release is also important for memory consolidation^12,17^ and promotes hippocampal neurogenesis^38^. However, during normal aging, lactate levels increase^39^ due to reduced oxidative phosphorylation from mitochondrial dysfunction. Considering aging is a risk factor for AD, mitochondrial dysregulation and the subsequent increase in cerebral lactate is postulated to instigate disease onset^40,41^. For example, CSF lactate levels are increased in AD patients^25^. While elevated hippocampal lactate levels are observed at 12 months and correspond with cognitive deficits in the APP/PS1 amyloidogenic AD mouse model^27^. At this same age in APP/PS1 mice, we reported elevated tonic levels of hippocampal glutamate^8,42^ that would contribute to these observed lactate levels.

Although basal and transient lactate were similar between sexes of C57BL/6J mice, we did observe increased transient amplitude in the CA1 and CA3 compared to the DG. A couple of factors may be responsible for these differences. While histological analysis regarding the number of cells within a hippocampal subregion varies, the DG is known to have a higher density of neurons^43^. The increased MCT density reduces the amount of lactate present in the extracellular space. Additionally, pyramidal neurons require significantly more energy substrates to maintain synaptic integrity than granule cells resulting in larger lactate transient amplitude in the CA3 and CA1. This suggests that bioenergetic disruptions do not affect all neuronal populations equally particularly during AD progression.

In male mice, Aβ_42_-evoked lactate release coupled to glutamate clearance was observed in all subregions, while this was only prominent in the CA1 of female mice. This suggests sexually dimorphic bioenergetics in the DG and CA3 with female mice relying on greater glucose utilization due to decreased glutamate coupled lactate release. The enhanced gene expression of glial LDHA and SLC16A1 as well as neuronal LDHB could be a compensatory mechanism to increase lactate production, transfer, and utilization to meet hippocampal bioenergetic demands. The upregulation of CHRNA7 gene expression in conjunction with soluble Aβ_42_ accumulation causes excessive activation during AD progression. These factors coupled with the concomitant glucose hypometabolism in AD challenges the ANLS to maintain the electrochemical gradient and leaves these regions particularly vulnerable to neurodegeneration. The translational implications provides a better understanding as to the increased AD prevalence in females.

Accumulation and deposition of Aβ_42_ containing senile plaques is the earliest known pathological feature associated with AD. But, therapies targeting clearance of aggregated amyloid are only purported to modify disease progression in a subset of clinical trial participants^44^. This may be a consequence of how amyloid accumulation alters bioenergetics during AD progression. We have previously demonstrated soluble Aβ_42_ elicits glutamate release through sodium dependent presynaptic activation of the α7nAChR^7^. This mechanism contributes to elevated hippocampal tonic and phasic glutamate levels prior to onset of cognitive deficits in APP/PS1 mice^8^. The current study demonstrates Aβ_42_ also elicits lactate release coupled to glutamate uptake as a possible means to maintain energy demands for enhanced synaptic signaling based on the ANLS. Furthermore, neuronal activity is known to modulate Aβ_42_ accumulation and aggregation suggestive of a vicious pathophysiological cascade^45^. During the initial phases of Aβ_42_ accumulation, astroglia might be able to sustain the enhanced energy substrate needs. However, amyloid deposition alters the morphology and function of astroglia. These reactive astrocytes have decreased expression of EAAT1 and 2 as well as glucose transporter 1^46^. This would diminish their bioenergetic capacity resulting in glucose hypometabolism and neuronal loss typified in later stages of AD progression more akin to cognitive deficits^47^.

The main source of oxidative substrates for neuronal energy demands is contested throughout the literature. Neurons can readily utilize both glucose and lactate as an energy substrate. But, vascular dysfunction and reduced cerebral blood flow are observed in AD^48^. The subsequent decrease in blood derived glucose forces neurons to rely on lactate. Additionally, synaptic glutamate release is heightened by Aβ_42_ activation thereby increasing ANLS as a means to meet the elevated neuronal energy demands. As soluble Aβ_42_ levels increase during AD progression, the elevated hippocampal activity^49^ makes larger pyramidal neurons particularly vulnerable to damage and is why they constitute a larger percentage of cell loss observed in post-mortem AD brains^50^. Prior to cell death, though, these damaged neurons undergo energetic inefficiency requiring additional substrates and creating competition for available resources with healthy neurons^41^. Eventually, the overutilization of energy substrates propagates neuronal dysfunction affecting neighboring brain regions. These changes in glutamate coupled lactate release may serve as an early biomarker for bioenergetic perturbations driving AD progression.

Limitations of the study are related to the specificity of the EAAT and MCT inhibitors. At the concentrations used, TBOA inhibits clearance of glutamate through both the glial EAAT1 and 2 and the neuronal EAAT3. Although > 90% of extracellular glutamate clearance is mediated by glial EAAT2 in the forebrain^10^, neuronal uptake is a contributing factor. Likewise, CHC at the concentrations studied inhibits transport of lactate across either glial MCT1 or 4 and neuronal MCT2. Thus, the nonspecificity of the inhibitors used in the present study suggests a role for glutamate coupled lactate release from either neurons or glia. Future experiments with selective inhibitors or knockdown of individual MCT are needed to definitely conclude the cellular localization Aβ_42_ stimulated lactate release.

## Conclusion

The present study provides evidence that soluble Aβ_42_ alters hippocampal lactate dynamics through mechanisms associated with glutamate release and clearance. Considering pathophysiological changes occur decades prior to dementia onset, the hyperexcitable neuronal networks would eventually culminate in bioenergetic dysfunction, neuronal death, and dementia progression. Hippocampal lactate dynamic differences may account for the increased incidence of dementia in females and further indicates a need for personalized patient care. Additional experiments are needed to dissect sexually dimorphic effects of lactate release in hippocampal subregions and their resulting impact on AD progression.

## Methods

All methods were conducted in accordance with relevant guidelines and regulations.

### Animals

Protocols for animal use were approved by the *Institutional Animal Care and Use Committee* at Southern Illinois University School of Medicine and in compliance with the ARRIVE guidelines. Male and female 3–6 month old C57BL/6J mice were obtained from Jackson Laboratory (Bar Harbor, ME; RRID:IMSR_JAX:000664) and allowed at least 1 week to acclimate to our animal facility before electrochemical measurements or gene expression. Mice were group-housed according to sex and genotype on a 12:12 h light/dark schedule with food and water available ad libitum*.*

### Chemicals

All chemicals were prepared and stored according to manufacturer recommendations unless otherwise noted. l-lactate oxidase (EC 1.1.3.2) was obtained from USBiological (Salem, MA; Cat: L3000-10) and diluted in distilled, deionized water to make a 1 IU/µl stock solution for storage at 4 °C. Sodium phosphate monobasic monohydrate, sodium phosphate dibasic anhydrous, 1,3 phenylenediamine dihydrochloride (mPD), sodium chloride, calcium chloride dihydrate, and H_2_O_2_ (30% in water) were obtained from Thermo Fisher Scientific (Waltham, MA). l-lactate sodium salt, bovine serum albumin (BSA), glutaraldehyde, dopamine hydrochloride (DA), l-ascorbic acid (AA), polyurethane (PU), tetrahydrofuran (THF), and N,N-Dimethylformamide (DMF) were obtained from Sigma-Aldrich Co. (St. Louis, MO). Human Aβ_1-42_ (Cat: AS-20276) and human scrambled Aβ_1-42_ (Cat: AS-25383) was obtained from AnaSpec (Fremont, CA) and stored at − 80 °C once reconstituted. CHC (Cat: 5029) and TBOA (Cat: 1223) was obtained from Tocris Bioscience (Minneapolis, MN) and stock solutions were stored at − 20 °C.

### Enzyme-based MEA

**Figure 4:**
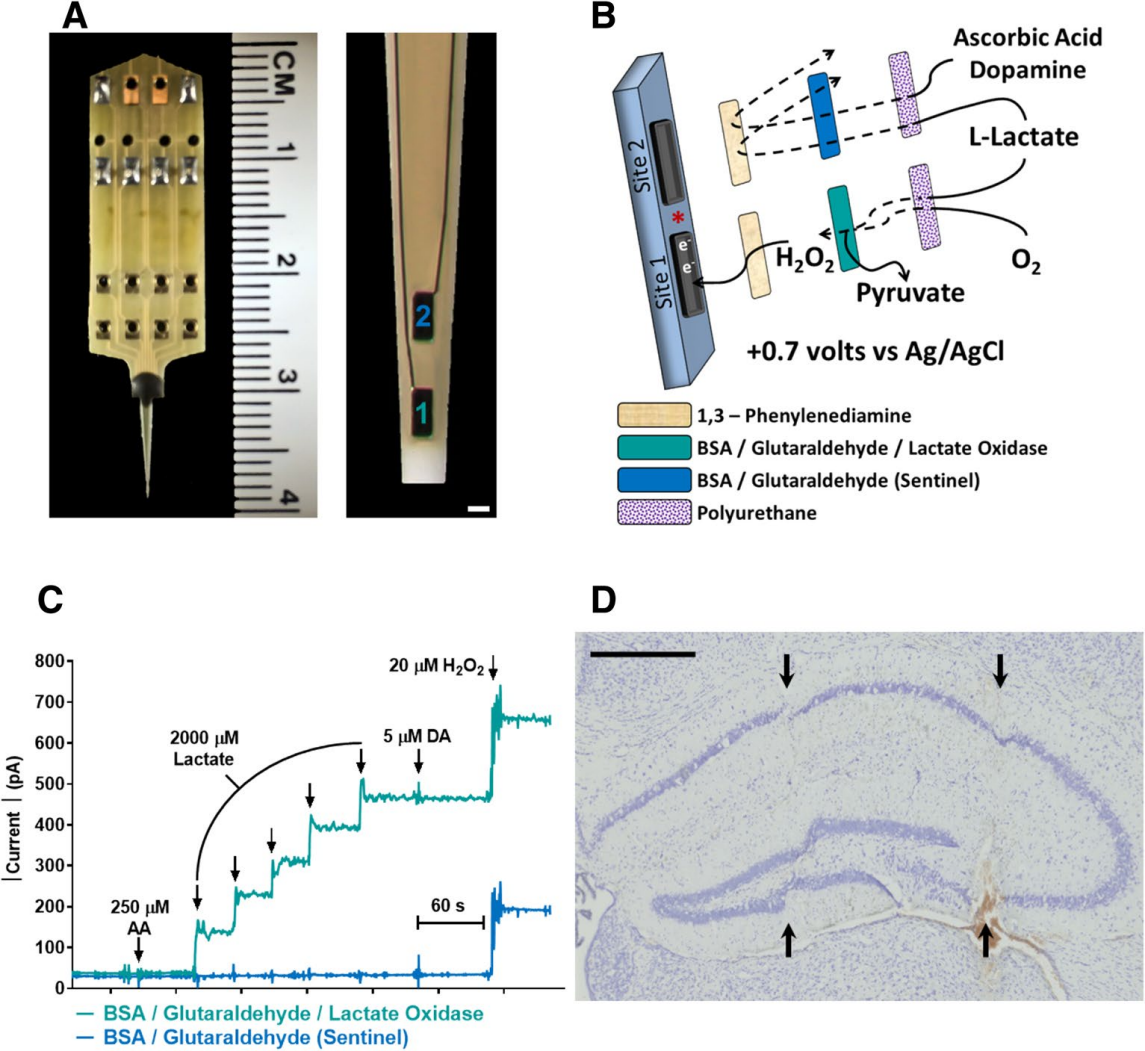
MEA selectivity for l-lactate hippocampal measurements. A photo of an R2 MEA with centimeter (CM) ruler for size comparison (**A**). A magnified view of the recording sites is shown to the right (scale bar = 50 µm). An exploded drawing of the coatings applied to the MEA for selective lactate measurements (**B**). A representative calibration curve showing lactate detection only on sites coated with lactate oxidase despite both recording sites oxidizing H_2_O_2_ (**C**). A representative cresyl violet stained hippocampus (**D**) with probe placement tracks for the CA1 and DG (left arrows) and CA3 (right arrows). Scale bar = 500 µm.

MEA with platinum (Pt) recording surfaces were obtained from Quanteon LLC (Nicholasville, KY; Fig. 4A) and made selective for l-lactate recordings as previously described^28,29^. One microliter of lactate oxidase stock solution (1 IU/µl) was added to 9 µl of a 1.0% BSA and 0.125% glutaraldehyde w/v solution and applied dropwise to a Pt recording surface. Lactate oxidase enzymatically degrades lactate to pyruvate and H_2_O_2_, the electroactive reporter molecule (Fig. 4B). The other Pt recording site (sentinel site) was coated with the BSA/glutaraldehyde solution that does not enzymatically generate H_2_O_2_. When a potential of + 0.7 V vs a Ag/AgCl reference electrode was applied to the recording surfaces, H_2_O_2_ is oxidized and the current generated from the two electron transfer is amplified and digitized by the Fast Analytical Sensing Technology (FAST) 16mkIII (Quanteon LLC) electrochemistry instrument.

### PU coating

Seventy-two hours after enzyme coating, MEAs were dip coated in a 2% PU solution dissolved in a 98% THF and 2% DMF. PU limits the amount of lactate reaching the enzyme layer without blocking diffusion of O_2_^51^.

### mPD electropolymerization

Twenty-four hours after PU coating, 10 mM mPD in 0.05 M phosphate buffered saline (PBS) was applied to each recording surface. FAST electroplating software applied a triangular wave potential with an offset of − 0.5 V, peak-to-peak amplitude of 0.25 V, at a frequency of 0.05 Hz, for 20 min. This created a size exclusion layer that restricts the passage of AA, DA, uric acid and 3,4-dihydroxyphenylacetic acid to the Pt recording surface^52^.

### Calibration

MEAs were placed in a stirred solution of 40.0 mL of 0.05 M PBS maintained at 37 °C using a recirculating water bath (Stryker Corp., Kalamazoo, MI) with a separate glass Ag/AgCl reference electrode (Bioanalytical Systems, Inc., West Lafayette, IN). Final beaker concentrations of 0.25 M AA, 2, 4, 6, 8, 10 M Lactate, 0.005 M DA, and 0.02 M H_2_O_2_ were used to determine MEA selectivity for lactate and create a standard curve for the conversion of current to lactate concentration (Fig. 4C).

### Micropipette assembly and intracranial application

Glass micropipettes (World Precision Instruments, Inc., Sarasota, FL) were pulled using a vertical micropipette puller (Sutter Instrument Co., Novato, CA) with an internal diameter of 12–15 µm. The micropipette tip was positioned between a pair of recording sites and mounted 100 µm above the surface. All solutions applied intracranially were diluted to the appropriate concentration in physiological saline and deoxygenated under nitrogen bubbling for a minimum of 20 min. Solutions were sterile filtered into the micropipettes. Fluids were pressure-ejected using N_2_ gas by a Picospritzer III (Parker-Hannafin, Cleveland, OH), with pressure (5–15 psi) adjusted to consistently deliver volumes between (25–75 nl) over 1–2 s intervals. Ejection volumes were measured using a stereomicroscope Luxo Corp., Elmsford, NY) with a calibrated eye piece reticule.

### Reference electrode

A Ag/AgCl reference wire was prepared by stripping the teflon coating from both ends of a silver wire (A-M Systems, Carlsberg, WA). One end was soldered to a gold-plated connector (Newark element14 Chicago, IL), while the other was placed (cathode) into a 1 M HCl bath saturated with NaCl that also contained a stainless steel counter wire (anode). Passing a + 9 V DC to the cathode versus the anode for 15 min deposited Ag/Cl onto the Ag wire.

### In vivo anesthetized recordings

Mice were anesthetized using 1.5–2.0% isoflurane (Abbott Lab, North Chicago, IL) in a calibrated vaporizer (Vaporizer Sales & Service, Inc., Rockmart, GA) and placed in a stereotaxic frame fitted with an anesthesia mask (David Kopf Instruments, Tujunga, CA). Body temperature was maintained at 37 °C with a water pad (Braintree Scientific Inc., Braintree, MA) connected to a recirculating water bath. A craniotomy was performed to access the dentate (DG; AP: − 2.0, ML: ± 1.0, DV: − 2.2 mm), CA3 (AP: − 2.0, ML: ± 2.0, DV: − 2.2 mm) and CA1 (AP: − 2.0, ML: ± 1.0, DV: − 1.7 mm) from Bregma^53^. The Ag/AgCl reference wire was positioned beneath the skull and rostral to the craniotomy thereby creating a two electrode system when the MEA was positioned in the region of interest.

Constant voltage amperometry (4 Hz) was performed with a potential of + 0.7 V vs the Ag/AgCl reference electrode applied by the FAST16mkIII. MEAs reached a stable baseline for 60 min before basal and transient lactate analysis followed by pressure ejection studies. The FAST software saved amperometric data, time, and pressure ejection events. Calibration data, in conjunction with a MATLAB (MathWorks, Natick, MA; RRID:SCR_001622) graphic user interface program was used to calculate basal, transient, and stimulus-evoked lactate. Five evoked lactate signals in each hippocampal subfield were averaged into a representative signal for comparison. Only one compound was pressure-ejected per hemisphere for each mouse.

### Cresyl violet staining

After lactate recordings, mice were euthanized with an overdose of isoflurane followed by rapid decapitation with sharp scissors. The brains were removed and placed in 4% paraformaldehyde for 24–48 h then stored in 30% sucrose. Twenty micron coronal sections through the hippocampus were obtained using a cryostat (Model HM525 NX, Thermo Fisher Scientific) and mounted on a glass slide. Slices were stained with cresyl violet and coverslipped. MEA placement was verified for each mouse (Fig. 4D).

### RT-PCR

A separate group of 3–6 month male and female C57BL/6J mice were euthanized according to the above procedure. The brain was extracted and the hippocampus was dissected on wet ice and stored at − 80 °C until processing. RNA was extracted from tissue by homogenization in Trizol Reagent and separated by centrifugation at 12,000×*g* for 15 min at 4 °C with chloroform. RNA was isolated by centrifugation at 12,000×*g* for 25 min at 4 °C in 100% isopropanol. The pellet was resuspended in RNAse free water and quantified using a NanoDrop Spectrophotometer. cDNA was synthesized using candidate primers (Integrated DNA Technologies; Table 2) and an iScript cDNA Synthesis Kit (Bio-Rad). Relative mRNA expression was analyzed by quantitative RT-PCR as previously described^54^ using the StepOne Real-Time PCR System (Thermo Fisher Scientific) and SYBR Green MasterMix (Bio-Rad) and using Ubiquitin-conjugating enzyme E2D2 (UBE2D2) as the internal housekeeping gene.

**Table 2:** mRNA primers.

Gene	Name	Forward primer	Reverse primer
BSG	Basigin	5′-CACCATGGCAGCCCTCTGGCCC-3′	5′-ATAGATAAAGATGATGGTAACCAACA-3′
CHRNA7	Cholinergic receptor nicotinic alpha 7 subunit	5′-CCTGCAAGGCGAGTTCC-3′	5′-CTCAGGGAGAAGTACACGGTGA-3′
LDHA	Lactate dehydrogenase A	5′-ATGCACCCGCCTAAGGTTCTT-3′	5′-GCCTACGAGGTGATCAAGCT-3′
LDHB	Lactate dehydrogenase B	5′-AGTCTCCCGTGCATCCTCAA-3′	5′-AGGGTGTCCGCACTCTTCCT-3′
SLC1A2	Excitatory amino acid transporter 2	5′-CTGGTGCAAGCCTGTTTCC-3′	5′-GCCTGTTCACCCATCTTCC-3′
SLC1A3	Excitatory amino acid transporter 1	5′-ACCAAAAGCAACGGAGAAGAG-3′	5′-GGCATTCCGAAACAGGTAACTC-3′
SLC16A1	Monocarboxylate transporter 1	5′-GGTGGGCAGTGTTAGTCGG-3′	5′-GATAGGACCTCCAGCATACATGA-3′
SLC16A7	Monocarboxylate transporter 2	5′-GGGCTGGGTCGTAGTCTGT-3′	5′-ATCCAAGCGATCTGACTGGAG-3′

Forward and reverse primers used for RT-PCR are listed for each gene.

### Statistical analysis

Prism (GraphPad Software Version 9, Inc., La Jolla, CA; RRID:SCR_002798) was used for all statistical analyses. For basal lactate and transient lactate release analysis, sexes and hippocampal brain regions were compared using a two-way ANOVA with Sidak’s post hoc analysis. For Aβ_42_ stimulated lactate release studies, sex and hippocampal subregions were analyzed independently with a one-way ANOVA followed by Dunnett’s post hoc analysis. A two-tailed t test was used to compare hippocampal mRNA expression levels between sexes. A single Grubb’s test was used to identify outliers. Bar graphs represent mean ± standard error of the mean (SEM) and the number of animals are indicated in the figure legends. Significance was determined at p < 0.05.

## Data Availability

Data is available upon reasonable request.

## Abbreviations

α7nAChR: Alpha 7 nicotinic acetylcholine receptor
Aβ: Amyloid-β
AA: Ascorbic acid
AD: Alzheimer’s disease
ANLS: Astrocyte-neuron lactate shuttle
BSA: Bovine serum albumin
CHC: 2-Cyano-3-(4-hydroxyphenyl)-2-propenoic acid
DA: Dopamine hydrochloride
DG: Dentate
DMF: N,N-dimethylformamide
EAAT: Excitatory amino acid transporters
FAST: Fast Analytical Sensing Technology
LDH: Lactate dehydrogenase
MCT: Monocarboxylate transporter
MEA: Microelectrode arrays
mPD: 1,3 Phenylenediamine dihydrochloride
PBS: Phosphate buffered saline
PU: Polyurethane
SEM: Standard error of the mean
TBOA: dl-threo-β-benzyloxyaspartic acid
THF: Tetrahydrofuran
